# Identification of Single Amino Acid Changes in the Rift Valley Fever Virus Polymerase Core Domain Contributing to Virus Attenuation *In Vivo*

**DOI:** 10.3389/fcimb.2022.875539

**Published:** 2022-04-28

**Authors:** Belén Borrego, Sandra Moreno, Álvaro López-Valiñas, Nuria de la Losa, Friedemann Weber, José Ignacio Núñez, Alejandro Brun

**Affiliations:** ^1^ Centro de Investigación en Sanidad Animal, CISA (Instituto Nacional de Investigación y Tecnología Agraria y Alimentaria/Consejo Superior de Investigaciones Científicas (INIA/CSIC)), Madrid, Spain; ^2^ Centre de Recerca en Sanitat Animal, Centre de Recerca en Sanitat Animal (CReSA) Institut de Recerca en Tecnologies Agroalimentàries (IRTA), Barcelona, Spain; ^3^ Institut für Virologie, FB10-Veterinary Medicine, Justus-Liebig-Universität Giessen, Giessen, Germany

**Keywords:** RVFV, viral polymerase, attenuation, live vaccines, reverse genetics, mutagenic drugs

## Abstract

Rift Valley fever (RVF) is an arboviral zoonotic disease affecting many African countries with the potential to spread to other geographical areas. RVF affects sheep, goats, cattle and camels, causing a high rate of abortions and death of newborn lambs. Also, humans can be infected, developing a usually self-limiting disease that can turn into a more severe illness in a low percentage of cases. Although different veterinary vaccines are available in endemic areas in Africa, to date no human vaccine has been licensed. In previous works, we described the selection and characterization of a favipiravir-mutagenized RVFV variant, termed 40Fp8, with potential as a RVF vaccine candidate due to the strong attenuation shown in immunocompromised animal models. Compared to the parental South African 56/74 viral strain, 40Fp8 displayed 7 amino acid substitutions in the L-protein, three of them located in the central region corresponding to the catalytic core of the RNA-dependent RNA polymerase (RdRp). In this work, by means of a reverse genetics system, we have analyzed the effect on virulence of these amino acid changes, alone or combined, both *in vitro* and *in vivo*. We found that the simultaneous introduction of two changes (G924S and A1303T) in the heterologous ZH548-RVFV Egyptian strain conferred attenuated phenotypes to the rescued viruses as shown in infected mice without affecting virus immunogenicity. Our results suggest that both changes induce resistance to favipiravir likely associated to some fitness cost that could be the basis for the observed attenuation *in vivo*. Conversely, the third change, I1050V, appears to be a compensatory mutation increasing viral fitness. Altogether, these results provide relevant information for the safety improvement of novel live attenuated RVFV vaccines.

## Introduction

Rift Valley fever virus (RVFV) is a prototypic phlebovirus species, classified in the Phenuiviridae family within the Bunyavirales order (Abudurexiti et al., 2019). The virus infects many wild and domestic ruminant species (sheep, goats, calves and camels), causing the homonymous Rift Valley fever (RVF), a disease characterized by high rates of abortion and neonatal mortality in domestic ruminants, that poses a major economic impact in low-income African countries. Humans near affected flocks may become infected by fomites or through bites of mosquitoes previously fed from viremic animals. Most infected humans usually develop flu-like symptoms, but more severe complications can develop in a low percentage of the infected population, leading to encephalitis or to a, usually fatal, haemorrhagic disease (Dungu and Bouloy, 2021). All these features make RVF a paradigm of disease for the One Health concept. RVF is widespread in Africa, where several outbreaks in the continent and in the Arabian Peninsula have been reported in the last decades. More recently, some countries surrounding the Mediterranean basin, officially free from RVF, have reported seropositive results in animals and humans, thus highlighting the risk of disease spreading (http://www.promedmail.org; http://www.afro.who.int). Therefore, RVF has been listed as a top priority disease for developing control measures in the following years (Mehand et al., 2018).

The RVFV genome consists of three single-stranded, negative-sense RNA segments [ssRNA(-)] differing in size [Small (S), Medium (M) and Large (L)]. The S segment encodes two proteins in an ambisense strategy: the 27 kDa nucleoprotein (N) that protects the viral RNA, and a 30 kDa non-structural protein, NSs, a polyfunctional protein considered the main virulence factor of the virus (Ly and Ikegami, 2016). Deletion or manipulation of NSs attenuates the virulence of the virus in immunocompetent hosts, thus becoming an excellent target for the generation of attenuated vaccines (Muller et al., 1995; Kalveram et al., 2011).

The M segment codes for the two viral envelope glycoproteins Gn and Gc, and for a non-structural 14kDa protein, NSm, with some role in modulation of apoptosis (Won et al., 2007), and viral attenuation (Bird et al., 2007). A 78k Da glycoprotein (P78) comprised by an unprocessed NSm-Gn polypeptide can be also synthesized, with unknown function, although modifying its expression levels can affect the efficiency of virus replication in macrophages and, subsequently, the virulence *in vivo* (Terasaki et al., 2021).

The L segment of bunyaviruses encodes the single L-protein, a multifunctional enzyme 2092 amino acid-long that includes a central RNA-dependent RNA polymerase (RdRp) domain responsible for replication and transcription of the viral RNA genome, an endonuclease domain (END) at the N terminus and a cap binding domain (CBD) at the C-terminal region (Reguera et al., 2010). This architectural and functional structure is shared by other negative-strand segmented RNA viruses such as arena- and orthomyxoviruses (Gerlach et al., 2015; Ferron et al., 2017). Although different in sequences, RdRps are the most conserved of the proteins encoded by RNA viruses, all of them displaying the canonical fold of the RdRp catalytic core, consisting of palm, fingers, and thumb domains, that harbours seven conserved motifs (A to G) involved in different steps of RNA replication. In these motifs, key conserved residues for different enzymatic functions have been identified (te Velthuis, 2014; Ferron et al., 2017; Venkataraman et al., 2018). Based on this high degree of sequence identity, the change of polymerase residues involved in key steps of viral replication has been proposed as a universal approach to obtain attenuated RNA viruses for vaccine purposes (Vignuzzi et al., 2008; Weeks et al., 2012; Yeh et al., 2020; Mori et al., 2021). Recently, the structure of the RVFV L-protein has been solved by cryo-EM, providing the precise location of most of these structural motifs and residues (Wang et al., 2021).

Due to the peculiar epidemiology of RVF, vectored by virus-infected mosquitos and highly dependent on climatic conditions, preventive vaccination against RVF is the main tool for an efficient control of the disease. Both live-attenuated and inactivated vaccines are available in Africa for veterinary use. While inactivated vaccines need booster doses, vaccines based on live-attenuated RVFV allow more successful immunization programs, but their use is usually limited because of risks associated to their residual virulence, especially for immune compromised hosts, or in pregnant sheep (Makoschey et al., 2016; Dungu et al., 2018; Dungu and Bouloy, 2021). On the other hand, a licensed human vaccine is not yet available in endemic countries, not even for high-risk groups exposed to RVFV infection during RVF outbreaks. A live-attenuated thermosensitive vaccine, MP12, obtained under selective mutagenic pressure (Caplen et al., 1985) proved to be safe in humans, with low-to-moderate side effects and induction of long-term antibody responses after a single dose (Pittman et al., 2016), but its application as human vaccine needs further improvement (Ikegami, 2019). A number of novel vaccines in development are based on the immunogenic glycoproteins delivered as subunit or by different platforms [reviewed in (Faburay et al., 2017; Ikegami, 2019)]. Although some of these approaches are providing promising results (Wilson et al., 2021), live attenuated vaccines remain as the strongest immune inductors in terms of duration and breadth of the immune response after a single dose inoculation, and their improvement in terms of safety has been the subject of intense RVF research in the past decade (Bird et al., 2011; Kalveram et al., 2011; Kortekaas et al., 2011; Wichgers-Schreur et al., 2020). The availability of RVFV reverse genetics systems allows designing novel rational attenuation strategies obviously conditioned by the previous identification and understanding of such attenuating determinants (Tercero and Makino, 2020).

In a former work we obtained a favipiravir-mutagenized, RVFV 56/74 virus variant (termed 40Fp8) that was highly attenuated *in vivo*, even in immunosuppressed A129 (IFNAR KO) mice (Borrego and Brun, 2020). The 40Fp8 variant carries 23 synonymous and 24 non-synonymous mutations, some of them located in protein domains likely involved in the attenuated phenotype. We have already shown that one change in the non-structural NSs protein, P82L, was a determinant of attenuation (Borrego et al., 2021). In this work we focused on three substitutions in the central region of the L-protein: G924S, I1050V and A1303T. Their location within defined polymerase domains as well as their conservation among RdRps from several phlebovirus species strongly suggested an important role in the activity of the protein and therefore in the attenuated phenotype of the virus. Our results show that the combination of two single changes, G924S and A1303T, is enough to strongly diminish the virulence of the wt rZH548 virus, while keeping its ability to induce an immune protective response.

## Materials and Methods

### Cells and Viruses

The cell lines used for this study were HEK293T (SV40 T-antigen transformed human embryonic kidney 293 cells, ATCC CRL-3216), BHK-21 (baby hamster kidney fibroblasts, ATCC CCL-10) and Vero (African green monkey kidney cells, ATCC CCL-81). All cell lines were grown as described (Borrego and Brun, 2020). Viruses generated and analyzed in this work are listed in **Table 1**, including those previously described and used as controls in the different assays: rZH548 (wild-type), a NSs-deleted virus expressing green fluorescent protein, named as rZH548ΔNSs::GFP (Moreno et al., 2020), and a ZH548 NSs-mutated virus with the amino acid substitution P82L, shortnamed as 2B7 (Borrego et al., 2021). Infections and titrations were performed as described (Borrego and Brun, 2020).

**Table 1:** rRVFVs generated and analyzed in this work, corresponding to the combinations of the 3 single positions selected within the L-protein (segment L), together with the different genotypes regarding NSs gene (segment S).

Mutant rRVF virus		924-1050-1303	Segment L	Segment S	Features
**#1**	Single mutants (SM)	**S-I-A**	(2788) _G924S	ΔNSs:gfp	NSs gene deleted (substituted by gfp) and single nucleotide substitutions in the L gene, alone or combined as indicated
**#2**		**G-V-A**	(3166) _I1050V		
**#3**		**G-I-T**	(3925) _A1303T		
**#4**	Double mutants (DM)	**S-V-A**	(2788/3166) _G924S/I1050V		
**#5**		**S-I-T**	(2788/3925) _ G924S/A1303T		
**#6**		**G-V-T**	(3166/3925)_I1050V/A1303T		
**#7**	Triple mutant (TM)	**S-V-T**	(2788/3166/3925)_ G924S/I1050V/A1303T		
**rA2**	L#5/S279	**S-I-T**	(2788/3925) _ G924S/A1303T	(279)_NSs[P82L]	Two nucleotide substitutions in the L gene (DM #5) and full-lenght NSs protein (mutated or wt as indicated)
**rB3**	L#5			wt	
**CONTROL VIRUSES** ^1^					
**C1**	rZH548	**G-I-A**	wt	wt	Virulence control
**G1**	rZH548_ ΔNSs:gfp		wt	ΔNSs:gfp	Attenuation control
**2B7**	rZH548-P82L		wt	(279)_NSs[P82L]	wt L (ZH548) and single nucleotide substitution in the NSs gene

First column shows the short name used for each virus along this study, the other columns indicate the main differential characteristics. The nucleotide positions in the corresponding RNA-segments where changes have been introduced are indicated in parenthesis, followed by the corresponding amino acid substitution(s) in the viral protein (in one-letter code). Numbering and sequences are according to DQ375403 RVFV segment L and NC_014395 RVFV segment S, strain “ZH-548”. Column 3 shows the resulting amino acid residues for each virus in the three positions of the L-protein under study (924, 1050 and 1303). ^1^ These viruses were included in the different assays as control or reference viruses, and have already been described. Deletion of NSs (rRVFV G1) renders the virus attenuated for immunocompetent mice, while being fully virulent in IFNAR KO mice (Bouloy et al., 2001); substitution at nucleotide 279 in segment S displayed by virus 2B7 renders a partially attenuated virus in Balb/c mice (Borrego et al., 2021).

### Infection in the Presence of Drugs

Favipiravir T-705 (Atomax chemicals Co. Ltd.) was dissolved in DMSO at a concentration of 100 mM, aliquoted and stored at -70°C. Ribavirin (Sigma R9644) was dissolved in water at a concentration of 50 mg/ml (205 mM), aliquoted and stored at -70°C. For use in the experiments, the solutions were thawed, and diluted in DMEM to reach the desired concentration indicated in each experiment. For infections in the presence of favipiravir, cells were preincubated with the drug overnight prior to infection, then the virus was adsorbed for 1 h to cells in absence of drug, and then infection continued in the presence of the drug. For infections in the presence of ribavirin, the drug was added to the cells after virus adsorption and maintained in the culture.

### Rescue of Recombinant Viruses

Recombinant ZH548 RVF viruses were rescued by means of a reverse genetic system (Habjan et al., 2008; Moreno et al., 2020). Briefly, the system is based on transfection of 5 plasmids into co-cultures of HEK293T and BHK-21 cells. The 5 plasmids consists of 3 plasmids providing viral genomic segments L, M and S (pHH21_RVFV_vL, pHH21_RVFV_vM and pHH21_RVFV_vS respectively) and 2 plasmids providing the viral polymerase L and the nucleoprotein N (pI.18_RVFV_L and pI.18_RVFV_N). In some transfections the plasmids providing the viral segment S corresponded to previously described mutant versions: a NSs gene-deleted expressing green fluorescent protein; and a single NSs mutant at nucleotide position 279 (Moreno et al., 2020; Borrego et al., 2021).

To generate the plasmids carrying the mutant L segments, the desired nucleotide changes were introduced in plasmid pHH21-RVFV-vL by PCR using the Q5^®^ Site-Directed Mutagenesis Kit (NewEngland Biolabs) following manufacturer´s protocols. The primers used, designed using the NEB online design software NEBaseChanger™, are shown in **Table 2**. On days 3, 5 and 7, transfected cell supernatants were harvested and inoculated onto Vero cells and screened for the presence of mutant virus by cytopathic effect (CPE) or by visualization of infection foci by green fluorescence. For those rendering positive results, further two passages were performed to generate a virus stock (p3) that was used in the different experiments.

**Table 2:** Primers used in this study.

Oligo	Nucleotide positions	Sequence 5’→3’	Use
L2788Fwd	2788-2804	(P)**A**GTCTGAGAGAGATCTA	Nucleotide change
L2788Rev	2768-2787	(P)TCCATGCTGTTGTTTCTTAA	
L3166Fwd	3166-3187	(P)**G**TTAGAGATGACTTTGTGATGG	
L3166Rev	3149-3165	(P)ATCAAGCTCCCGATGAC	
L3925Fwd	3915-3935	TCTCTTCAAA**A**CTATCACCAG	
L3925Rev	3897-3914	TTAAATCTGAAACCTCCC	
RdRp Fwd	2701-2723	GCTATCCAGAAGTTTGAGGATTG	PCR and sequencing of RdRp core
RdRp Rev2	4055-4077	TAGAGAGGAGCTAAGAATTAGAG	

Nucleotide changed to introduce the corresponding mutation is bold-typed. The correspondence between nucleotide changed at the indicated position within the segment L and amino acid substitution in the L-protein is as follows: change G2788A leads to Gly924Ser, change A3166G leads to Ile1050Val, and change G3925A leads to Ala1303Thr. Sequences are based on the sequence DQ375403.1 corresponding to Rift Valley fever virus strain ZH-548 segment L.

### Sequencing Analysis

The amino acid positions under study were analyzed by automatic Sanger-sequencing of the corresponding purified amplicons. Briefly, RNA was extracted from the supernatants of infected cells or blood samples, and amplicons corresponding to the NSs ORF and to the RdRp core (nt positions 2701-4077) were obtained by RT-PCR, using primers as described [(Borrego et al., 2021), **Table 2**].

Polymerase fidelity was tested by next-generation sequencing (NGS) made on a 1100 nt amplicon fragment corresponding to the amino-terminal half of Gc glycoprotein from passage 3 viruses after an additional passage on Vero cells at a multiplicity of infection (moi) of 0.1 (Borrego et al., 2019). RT-PCR was performed on RNA extracted from supernatants collected at 72 hpi using SuperScript IV Reverse Transcriptase (Invitrogen) and Phusion High-Fidelity DNA polymerase (Finnzymes). Amplicons were purified and sequenced as follows: libraries were created using the NEBNext^®^ Ultra™ II DNA Library Prep Kit and sequenced with Truseq instrument, using protocols and reagents from Illumina (Illumina^®^, San Diego, CA, USA) at “Unidad Genómica FPCM” (Madrid, Spain).

The quality of the reads was assessed by FastQC (v 0.11.8) (Andrews, 2010). Reads with a quality lower than 30 were removed using Trimmomatic (v0.39) (Bolger et al., 2014). Subsequently, Burrows-Wheeler Aligner (BWA) was used to align reads of each sequenced sample against the corresponding region of the pHH21_RVFV_vM plasmid used for virus rescue (Li and Durbin, 2009). Those reads unmapped, with a quality map lower than 30, were removed using Samtools (v.0.39) (Danecek et al., 2021). In addition, PCR duplicates were also removed with the Picard “MArkDuplicatesSpark” and base recalibration was performed with “BaseRecalibrator”, both included in GATK4 (v4.1). Finally, variants of each sample were noted in a variant calling file (VCF) generated with LoFreq software (v2.1.5) using default parameters (Wilm et al., 2012). A variant was considered when following these requirements: mapping quality > 30, minimum coverage depth of 100, alternative base supported by at least 10 reads, and p value per change found <0.01 (Lopez-Valinas et al., 2021). Finally, the nucleotide diversity (π) was calculated in each sample with SNPGenie software (Nelson et al., 2015).

### Animal Inoculation and Sampling

*In vivo* studies were done using either 129Sv (wt) or A129 (IFNAR KO) mice bred in our facilities at Department of Animal Reproduction (INIA/CSIC); mice were housed in biosafety level 3 (BSL-3) animal facilities at CISA before use. The composition of the groups in terms of sex and age was dependent on their availability and is indicated in each experiment. Animals were equally distributed into groups of 5-7 and inoculated intraperitoneally with the corresponding viruses at the indicated doses. Development of disease was evaluated daily over 3 weeks (circa) in terms of morbidity (weight, fur, activity, posture) and mortality (Borrego et al., 2021). At 72 h after infection blood samples were taken by submandibular puncture and tested for viral RNA by RT-qPCR (Mwaengo et al., 2012; Moreno et al., 2020) to monitor viremia, while serum samples to be used in antibody assays were collected at 2 weeks pi or at the end of the experiment. All experimental procedures involving animals were performed in accordance with EU guidelines (directive 2010/63/EU), and protocols approved by the Animal Care and Biosafety Ethics’ Committees of INIA and Comunidad de Madrid (permit codes CBS 2017/15 and PROEX 192/17).

### Antibody Assays

Antibodies against NP were detected by an in-house ELISA and RVFV neutralizing antibodies in a plaque reduction neutralization assay (Borrego et al., 2021). Anti-NP titers are expressed as the last dilution of serum (log10) giving an OD reading at 450 nm over 2X the background value; neutralization titers are expressed as PRNT80, i.e., dilution of serum (log10) rendering a reduction of infectivity of 80%.

### Statistical Analysis

GraphPad Prism version 6.0 (GraphPad Software, San Diego, CA) was used for statistical analyses. Tests performed are indicated in the corresponding experiments. Differences were considered statistically significant when p < 0.05.

### 3D Structures

The PyMOL Molecular Graphics System, Version 4.6 was used to visualize the L-protein structure.

## Results

### Generation by Reverse Genetics and *In Vitro* Analysis of NSs-Deleted rRVFV L-Mutants

With respect to the parental virulent RVFV-56/74 strain the attenuated 40Fp8 virus carries nucleotide substitutions in the three genomic segments. To analyze their role in attenuation we first focused on the L segment, since it codes for the L-protein, the viral RNA-dependent RNA polymerase (RdRp) known to be the target of favipiravir, the drug used for 40Fp8 selection. Out of the 7 amino acid changes identified in the 40Fp8 L-protein, we selected for deeper analysis 3 substitutions (G924S, I1050V and A1303T) located in the predicted catalytic RdRp core (Borrego and Brun, 2020). We planned to generate 7 recombinant viruses (rRVFV mutants #1 to #7) corresponding to all possible combinations of these 3 mutations: 3 single mutants (SM), 3 double mutants (DM), and the triple mutant (TM) (**Table 1**). These rRVFVs were generated using a reverse genetics system based on the Egyptian lineage isolate RVFV-ZH548 (Habjan et al., 2008). We first obtained plasmids for the L-single mutants, by introducing the corresponding nucleotide substitution into the plasmid pHH21_RVFV_vL. The single-mutant-plasmids were then used as templates for the obtention of those carrying two and three substitutions successively. The correctness of the resulting plasmids was checked in all cases by Sanger sequencing.

For the rescue of the desired rRVFVs, co-cultures of HEK293T and BHK-21 cells were transfected in duplicates. Since sequence alignments of RVFV isolates and phleboviruses indicated that residues G924 and A1303 were extremely conserved (Borrego and Brun, 2020), we expected some growth deficiency of viruses carrying such changes, if rescued. Therefore, in order to provide an easier monitoring of the procedure in this first attempt, we used for transfection an S-segment-plasmid in which the NSs protein gene had been deleted and replaced by a reporter gene (EGFP) (Habjan et al., 2008; Moreno et al., 2020). At the same time, viruses corresponding to rZH548 wt and rZH548 ΔNSs::gfp were generated to be used as controls in the forthcoming assays and were short named C1 (control) and G1 (green) respectively.

Supernatants harvested at different times post-transfection were inoculated onto Vero cells. Not surprisingly, cytopathic effect (CPE) was difficult to detect in some cases but green fluorescence allowed us the visualization of rescued L-mutants-rRVFVs. While SM #2 or DM #6 were easily obtained in the first attempt, SM #1 and DM #5 needed 6 and 5 attempts, respectively.

**Figure 1:**
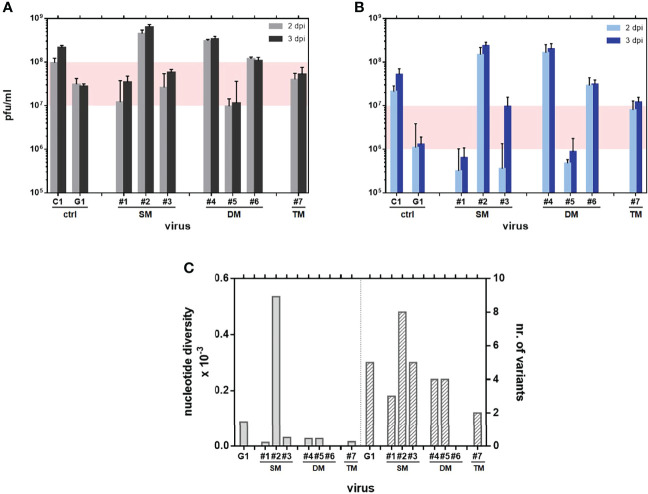
Kinetics of growth in mammalian cells: Vero **(A)** and HEK293T **(B)** cells were infected at a MOI of 0.01 with the indicated rRVF viruses (**Table 1**). Supernatants collected at 48 and 72 hpi were titrated onto Vero cells by a plaque assay. Experiments were performed in duplicate. Data shown correspond to a single representative assay. The range of final yield between both wild-type polymerase-control viruses C1 and G1 has been shaded. **(C)** Nucleotide diversity bars per sequenced sample (left) and total number of variants annotated per sample (right). No data could be retrieved for DM#6.

Viruses were grown in Vero cells for 3 consecutive passages. After confirming the presence of the desired mutations (and no other changes leading to amino acid substitution in the surrounding region spanning nucleotide positions 2701-4077), viral growth (yield and kinetics) was analyzed both in Vero and in HEK293T cells (**Figures 1A, B**).

In Vero cells the mutant viruses did not show a strong deficiency in growth, rendering similar titers to those of the control virus G1 also lacking the NSs protein. There were however slight differences among them: DM #5 showed the lowest yield (10^7^ pfu/ml), only slightly below that of G1. On the other hand, some mutants showed unexpected high final yields, close to that rendered by the wt virus C1 (10^8^ pfu/ml), or even higher, such as DM #4 and SM #2. Regarding plaque phenotype, all rescued viruses rendered a typical, less cytolytic, diffuse plaque on Vero cell monolayers associated to the NSs deletion. Viruses SM#1, SM#3 and TM#7 showed particularly tiny plaques, hampering visualization and quantification of plaques (not shown).

Yield differences were enhanced when growing in HEK293T cells. Since these cells are able to produce type-I IFN in response to viral infection, we used them as a system for a preliminary evaluation of the sensitivity of viruses to IFN. Our control viruses G1 and C1 showed the expected profiles: C1 (rZH548 wt virus) displays a fully functional NSs protein able to counteract the cellular IFN system therefore reaching high viral titers (around 5x10^7^ pfu/ml). In contrast G1, because of the deletion of the NSs gene, cannot block the cellular IFN system resulting in a reduced yield (1x10^6^ pfu/ml). Surprisingly, even though all L-mutants lack NSs expression as G1 virus, different growth patterns were found. Only mutants SM #1 and DM #5 showed the expected IFN-sensitive G1-like profile with lower viral yields: (6-9 x10^5^ pfu/ml). In contrast, mutants SM #2, DM #4, DM #6 and TM #7 (all of them carrying the change at position 1050, alone or combined) were not affected by the lack of NSs and rendered titers equivalent to or even higher than that of the wt virus C1 (10^7^ – 10^8^ pfu/ml). SM #3 showed a hybrid phenotype, appearing IFN-sensitive at the first time of infection analyzed (day 2) but subverting this effect by day 3. Except for this peculiar kinetics, every virus maintained the pattern of low or high yield in both cell types, suggesting that regardless any detrimental effect in production due to the lack of NSs, the introduction of some changes in the RdRp might confer growth advantage increasing viral production. In particular, the substitution I1050V, alone or combined, introduced in SM #2, DMs # 4 and #6 and TM # 7, strongly improved viral growth.

We also explored whether changes under study had some effect on the fidelity of the corresponding mutant polymerases. Mutant viruses #1 to #7 were subjected to an additional passage in Vero cells, and a 1,100 nt-long region of the amino-terminal half of glycoprotein Gc was analyzed by NGS. Nucleotide diversity (π) of each sample population was calculated as described in materials and methods. G1 virus was included to provide the data of basal variability associated to the wt polymerase. The values of nucleotide diversity displayed by almost all mutants were below the one displayed by G1 (**Figure 1C**), suggesting that the corresponding polymerases rendered viral populations with a reduction in variability between 6- and 2-fold respectively. In contrast, SM#2 showed a strikingly higher nucleotide diversity, with a 6-fold increase in variability. Unfortunately, no data could be obtained from the sample corresponding to DM#6.

### Infectivity of ΔNSs L-Mutants in Immunodeficient A129 Mice

In order to evaluate the effect of the changes introduced in their infectivity, the ΔNSs L-mutant viruses were inoculated into A129 IFNAR KO mice. Because of their sensitivity to viral infections, this strain of mice has been widely used in immunity and vaccination studies for a number of viruses, including RVFV (Lorenzo et al., 2015; Clarke and Bradfute, 2020; Zivcec et al., 2020). A control group was inoculated with G1, the ΔNSs-rRVFV carrying a wt L-protein. This virus is attenuated due to the lack of NSs expression, but A129 IFNAR KO mice are extremely sensitive to ΔNSs viruses succumbing shortly after challenge (Bouloy et al., 2001). Animals received 500 pfu of each virus by the IP route and were monitored daily for the appearance of signs of disease and percentage of survival. Results are summarized in **Table 3**.

**Table 3:** Survival, MST (median survival time, i.e., time at which fractional survival equals 50%) and statistically significance in IFNAR KO mice (n= 5) inoculated *via* IP with 500 pfu of the indicated viruses.

	IFNAR Mice (sex)	rΔNSs-RVF virus	MST	Log-rank (Mantel-Cox) test vs G1
Expt 1	Male	G1	2	–
		SM #1	3	p=0.0158
		SM #2	2	ns
		SM #3	3	p=0.0143
		DM #4	3	ns
		**DM #5**	**4**	**p=0.0047**
		TM #7	3	p=0.0143
				
Expt 2	Female	G1	3	—
		SM #2	3	ns
		SM #3	3	ns
		**DM #5**	**4**	**p=0.0207**
		DM #6	3	ns

Experiments were carried out independently depending on the sex of the animals. Results were analyzed by the Log-Rank (Mantel-Cox) test. ns, non-significant differences. Results obtained with the rRVFV double mutant #5 are bold- typed to highlight their statistically significant differences.

A first trial was conducted with 11–18-week-old males. Even though there were no survivors, with 100% animals dying in a very short period after inoculation (2-4 days), some delay in disease onset was observed (not shown), as well as an increase in median survival time (MST) for all L-mutant viruses except for SM#2, bearing the I1050V substitution. Animals inoculated with this mutant rendered a MST of 2 days, similar to those receiving the G1 control virus. The other groups rendered slightly longer but statistically significant survival times, thus suggesting some level of attenuation associated to the changes introduced in positions 924 and/or 1303. The best results in terms of extended survival times were obtained with the double mutant #5 (carrying both changes G924S and A1303T), with an MST of 4 days compared to 2 days in the control group.

A second trial was performed with 15 week-old females where the double mutant #6, not included in the first assay, was tested together with the corresponding single mutants #2 and #3, as well as G1 as reference control. DM #5 was also included to confirm the higher attenuation observed in the male experiment. Again, all animals died before day 5, and results showed a statistically significant increase of MST in animals inoculated with DM #5 (4 days versus 3 in the control group), thus confirming the attenuating effect of the combination of both amino acid substitutions in positions 924 and 1303. In this experiment carried out with female mice, known to be less susceptible to RVFV infection than male (Tokuda et al., 2015), none of the other viruses tested showed significant differences compared with the reference virus. Therefore, the combination of changes displayed by double mutant #5 was selected for subsequent studies. Unfortunately, due to the rapid death of the animals, no samples were obtained from these experiments that could allow further studies.

### Rescue of rRVFV L-Mutants and Preliminary Characterization in Cell Culture

The seven L-mutant viruses obtained lacked NSs expression. To establish a better correlation between the changes introduced in the L-protein and the virulence phenotype, we decided to generate new recombinant viruses in a wild-type context. Based on the above-mentioned results, we selected the combination of changes that rendered the best results in terms of attenuation, i.e., G924S and A1303T (double mutant #5). The rRVFV rescue procedure was carried out as described, with cells transfected with the collection of five plasmids including the L-plasmid used to generate DM #5 that carries the two selected mutations (at nucleotide positions 2788 and 3925). Two sets of transfections, each one performed in 4 replicates, were carried out in which different S-segment plasmids were used. In the first one, we used plasmid pHH21_RVFV_vS to obtain a ZH548/wt NSs protein. In the second one, we used a mutated version of the S-segment plasmid carrying a change in nucleotide at position 279. This change leads to the P82L substitution in the NSs protein, already identified as attenuator in Balb/c mice (Borrego et al., 2021). We were interested in exploring the effect on viral attenuation of the combination of these substitutions located in 2 different genomic segments.

**Figure 2:**
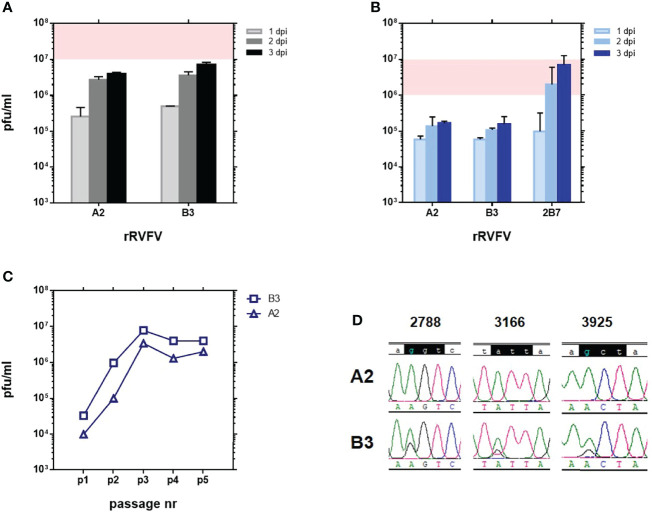
Growth in cultured cells of RVFVs rA2 and rB3 carrying substitutions G924S & A1303T in the RdRp. Viral yield at 24, 48 and 72 hpi after infection at a MOI of 0.01 in **(A)** Vero and **(B)** HEK293T cells. Although coming from an independent experiment, a shade has been depicted to allow for a comparison with results shown in **Figure 1**. In HEK293T infections, rZH548-P82L virus 2B7 was included for a comparison. Experiments were performed in duplicate. **(C)** Titer of supernatants collected at 72 hpi in serial passages (1 to 5) on Vero cells at a MOI of 0.01. Viral yields were determined by a plaque assay on Vero cell monolayers. **(D)** Sequence analysis of the RT-PCR product of viral RNAs recovered after 5 passages on Vero cells. The three nucleotide positions under study, 2788, 3166 and 3925, are shown. In the upper line, the wt sequence (rZH548) on the codons corresponding to residues 924, 1050 and 1303 is highlighted.

Recombinant rZH548 viruses were rescued in 1 out of the 4 replicates in transfections including the wt S plasmid, and 3 out of 4 using the mutant S-plasmid. Viruses were grown in Vero cells for 3 passages and the correct sequence of the introduced changes was checked. The rescued viruses were named respectively as rB3 [double mutant at RdRp (G924S & A1303T)] and rA2 [triple mutant at RdRp (G924S & A1303T) & NSs (P82L)] (**Table 1**). Viruses recovered after 3 passages were assayed for growth kinetics and yield in Vero cells. Although final titers were about one log unit lower than those reached by the wt virus (see **Figure 1A**), rA2 and rB3 viruses did not reveal any strong deficiency in growth, since final titers reached a value of 4-7x10^6^ pfu/ml (**Figure 2A**). On Vero cell monolayers plaques were smaller than those obtained with the rZH548 though well-defined and clear (not shown).

Growth of rRVFV-A2 and -B3 on HEK293T cells was also analyzed. For a comparison, in these assays we included an rRVFV previously obtained that carries the substitution P82L introduced in rA2. This change, although leading to a mutant NSs, does not affect viral growth in these IFN-competent cells. The virus, RVFV rZH548-P82L, was short-named as 2B7 (Borrego et al., 2021) (**Table 1**). Results with the 2B7 virus confirmed the ability of this NSs-mutant virus to grow efficiently in HEK293T cells. Surprisingly, both rA2 and rB3 mutant viruses showed the same IFN-sensitive pattern, with flat kinetics and low yields, even though their respective NSs, wt (B3) or mutant (A2), were expected to efficiently counteract the IFN-cellular system (**Figure 2B**). This pattern of growth in HEK293T cells was similar to that observed in some of the L-mutant ΔNSs viruses analysed previously (see **Figure 1B**), suggesting that besides the NSs phenotype (deleted, mutant or wt), changes in the L-protein might also affect the growth in HEK293T cells. In particular, the combination of changes selected, G924S and A1303T, seemed to have a dominant negative effect.

### Analysis of the Genetic Stability of rA2 and rB3

In order to assess the genetic stability in cell culture of the changes introduced in the rescued viruses, two further passages at a moi of 0.01 were performed on Vero cells and the sequences again checked. For these successive passages titers and growth remained consistent for both viruses (**Figure 2C**). After five passages (p5) rA2 maintained the changes introduced in the two nucleotide positions mutated, 2788 and 3925, whereas rB3 p5 showed traces of reversion in both positions and also some heterogeneity in position 3166 (**Figure 2D**). This change, which led to the substitution I1050V, was the third position found to change in the RdRp core of our 40Fp8 vaccine virus (Borrego and Brun, 2020) and analyzed in our first assays too, where results obtained with SM #2 suggested that it could provide some growth advantage (**Figure 1**). No other changes were found in the L- region analyzed (PCR amplicon corresponding to nucleotide positions 2701-4077). Because of these results, further assays were performed with viruses recovered after only three passages.

### Effect of the Amino Acid Substitutions in Viral Resistance to Mutagenic Drugs

**Figure 3:**
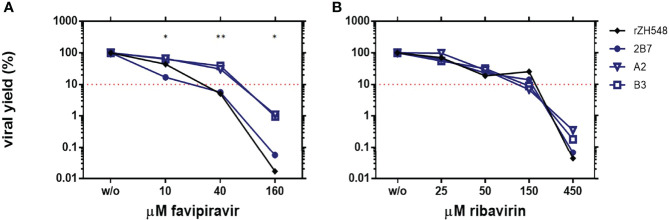
Susceptibility of rRVFVs to mutagenic drugs in Vero cells. Reduction of viral yield in the presence of different concentrations of the mutagenic drugs favipiravir **(A)** and ribavirin **(B)**. Vero cells were infected by duplicate at a MOI of 0.1 with the rZH548_L mutant viruses under study rA2 and rB3, and also with virus 2B7 displaying the substitution NSs[P82L] also present in rA2, and with the corresponding wt virus rZH548. Supernatants were collected at 72 hpi and titrated by plaque assay, and percentages of viral titers upon treatment were calculated. Geometric mean of the values obtained in at least two independent experiments is represented. Percentages of viral yield are represented as log10 in order to highlight the differences at the highest concentrations tested. The depicted dot-line corresponds to a reduction of 90% in viral yield. For statistical analysis each L-mutant RVFV was compared to its corresponding L-wt virus: rA2 vs 2B7 and rB3 vs rZH548. *p < 0,05; **p < 0,005 (multiple T-test, Holm-Sidak method).

Out of the three residues under study, residue 924 is located within motif F, a highly conserved motif of the RdRp involved in resistance to favipiravir (Abdelnabi et al., 2017; Goldhill et al., 2018). To test whether the change G924S could confer some degree of resistance to this drug, we analyzed viral yields of rA2 and rB3 in the presence of different concentrations of favipiravir and compared them to those obtained with rZH548 (C1), the virus carrying a wt L sequence and therefore “fully” susceptible to the drug. As in previous experiments, virus 2B7 was included for a comparison. As shown in **Figure 3A**, all the concentrations tested decreased virus yield, although reduction was much higher for the two viruses carrying a wt polymerase, C1 (rZH548) and 2B7. For these two viruses, a concentration of 40 µM favipiravir led to a reduction of viral yield over 90%, while at this concentration both rA2 and rB3 viruses still rendered about 30-40% of the production in the absence of the drug. Moreover, at 160 µM favipiravir the yield of L-mutant viruses remained in circa 1%, while for wt L- viruses was below 0.06%. These results indicated a significant difference in susceptibility to the drug linked to those L changes, although the mutant viruses remained susceptible to high concentrations of the drug.

In an analogous experiment, we tested whether these substitutions had also an effect on the susceptibility to ribavirin, another mutagenic drug targeting the viral polymerase. In contrast with results obtained with favipiravir, the curves representing how viral yield was affected on the presence of the drug showed a very similar profile for the four viruses, suggesting that the L-substitutions under study did not lead to statistically significant differences in the susceptibility to ribavirin of the corresponding polymerases/viruses (**Figure 3B**).

### Infectivity Assays in Immunodeficient A129 Mice

As previously stated, A129 IFNAR KO mice constitute an extremely sensitive system for assessing the degree of virus attenuation; therefore, the new rRVFVs ZH548_ B3 and _A2 were inoculated into 15-week-old IFNAR females. For each virus, 2 doses of inoculum, low and high (10 and 500 pfu, respectively) were tested. As a comparison, groups inoculated with the attenuated G1 virus (Lwt/ΔNSs) were included. Animals were monitored daily for 2 weeks to assess disease development and survival; on day 3 blood samples were taken to analyze viremia. The experiment was carried on until day 16, when survivors were euthanized and samples were taken for assessment of development of antibodies against viral proteins.

**Figure 4:**
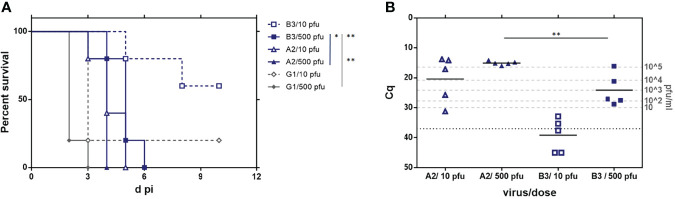
Analysis of the *in vivo* pathogenicity of the rZH548-L mutant viruses in IFNAR KO mice. 15-week-old female mice (n = 5) were inoculated with 10 or 500 plaque-forming units (pfu) of the RVFVs rA2 and rB3. G1 (rZH548ΔNSs::GFP) was included as reference control. **(A)** Survival rates. **(B)** Viremia determined by RT-qPCR on EDTA-blood samples collected at day 3 pi. Samples giving a Cq (quantification cycle) value under the detection level of the assay (37) are arbitrarily represented as 45. The correlation of Cq data with pfu equivalents (Moreno et al., 2020) is indicated in the right Y axis. Statistical analysis was only performed on results obtained from the groups inoculated with 500 pfu. The corresponding survival curves analyzed by log-rank (Mantel-Cox) rendered p values of p = 0.0047 (A2 vs G1); p = 0.0016 (B3 vs G1) and p = 0.0145 (A2 vs B3). *p < 0,05; **p < 0,005.

There were no survivors in the groups that received 500 pfu, regardless the virus inoculated, although some statistically significant differences were observed (**Figure 4A**). Mice inoculated with the G1 virus became ill and died very quickly (80% at day 2, MST = 2), avoiding the collection of samples. In groups A2 and B3 the first deaths occurred later (day 3 and day 4 respectively), leading to the corresponding increase in survival times (MST = 4 days for A2, and 5 days for B3). In the groups inoculated with 10 pfu, there were some survivors at the end of the experiment (d16): n = 1 in group G1, and n = 3 in group B3. However, when serum samples from these animals were analyzed by neutralization and anti-N ELISA, antibodies were only detected in one mouse belonging to group B3, suggesting that these survivors corresponded mainly to non-infected animals, probably due to infection failure with the low dose of 10 pfu (not shown). Therefore, these groups were not considered for the statistical analyses.

Survival results correlated with viremia levels at d3 pi detected by RT-qPCR (**Figure 4B**). Cq values of animals within the group B3/10 pfu were very close or even below the detection level of the assay (Cq 37.0). In all other samples, viral RNA was clearly detected, although the mean viral load showed differences between the different groups. Animals inoculated with only 10 pfu of rA2 showed a range of Cq between 13.8-31.2 (mean 20.4), while those receiving 500 pfu rendered very similar Cq values around 15.08, equivalent to a high viral load of 10^5^ pfu. In contrast, mice inoculated with 500 pfu of rB3 showed values in the range between 16.1-28.8, rendering a statistically significant lower Cq mean, 24.1 (unpaired t-test, p=0.0057).

These results confirm that the two substitutions introduced in the viral RdRp have an effect on virulence, although the provided attenuation was not enough to make these viruses safe for immune deficient animals, where they were able to replicate successfully and cause death. Compared to the deletion of NSs, these two substitutions seemed to provide a higher level of attenuation, as presumed from the MST mentioned data obtained in the groups inoculated with 500 pfu: 2 days in the G1 (ΔNSs) control versus 4 (rA2) or 5 days (rB3). Interestingly the viral loads obtained in these groups, as well as the survival data and morbidity profile (not shown) would suggest that for A129 mice, rRVFV_B3 is more attenuated than _A2, although this observation needs to be further confirmed.

The detection of viral RNA in 3 dpi-blood samples allowed us to explore the stability *in vivo* of the mutations introduced. We performed RT-PCR on the RNAs to amplify the region corresponding to the RdRp core and the amplicons were purified and Sanger-sequenced. Samples corresponding to animals inoculated with rRVFV_A2 were also used for amplification and sequencing of the NSs ORF. No changes were found in this region, with all samples maintaining the sequence corresponding to the inoculated mutant virus NSs [P82L].

RdRp sequencing results are shown in **Table 4**. In the groups inoculated with rRVFV_B3, RdRp core was only successfully amplified from samples recovered from animals that received the high dose, 500 pfu (B1 to B5). Two of these five samples maintained the sequence of the inoculum (mutant version: S924 & T1303), while the other three showed the total reversion to the ZH548 wt sequence in both positions (G924 & A1303), without any trace of the nucleotide displayed by the original inoculum at the corresponding position. Surprisingly, in these three samples the nucleotide at position 3166 was found to have changed, leading to the substitution I1050V, the same change appearing after 5 passages of rB3 in Vero cells. Thus, these “revertant” sequences recovered from IFNAR mice corresponded to a “hybrid” virus between ZH548 wt (G924 & A1303 residues) and mutant 40Fp8 (V1050), i.e., sequence G-V-A in the three positions under study, like SM #2 (**Table 1**).

**Table 4:** Amino acid (one-letter code) found in the three positions of the L-protein under study (924, 1050 and 1303) as deduced from the corresponding nucleotide sequence.

		Aa 924	Aa 1050	Aa 1303
Reference viruses	rZH548 wt	G	I	A
	rZH548_Lmut inoculum	S	I	T
**Viral RNAs recovered from IFNAR KO mice inoculated with rZH548_Lmut (rA2/rB3)**	A1	S/G	I	T
	A2	S/G	I	T
	A3	S	I	T
	A4	S	I	T
	A5	S/G	I	T
	A6	S	I	T
	A7	G	V	T/A
	A8	S	I	T
	A9	S	I	T
	A10	S	I	T
	B1	S	I	T
	B2	S	I	T
	B3	G	V	A
	B4	G	V	A
	B5	G	V	A
	**RVFV 40Fp8 mutant**	**S**	**V**	**T**

For positions showing a nucleotide mix that led to a change in the corresponding codon, the two possible amino acids are indicated, with slash. Samples recovered are coded as A (animals inoculated with rRVFV_A2) or B (animals inoculated with rRVFV_B3). Each sample correspond to an individual mouse. Numbers 1 to 5 correspond to animals receiving the high dose of virus, 500 pfu; numbers 6 to 10, those inoculated with the low dose, 10 pfu (only for A2 group).

Regarding the groups inoculated with rRVFV_A2, all 10 samples recovered from both low and high doses rendered positive RdRp core amplicons. Changes to reversion were also found on the two target positions but, as shown after 5 passages in cultured cells, rA2 appeared to be more stable despite the higher levels of replication suggested by the viral loads (**Figure 4B**). One only sample, corresponding to an animal inoculated with 10 pfu, showed a total reversion at position 924, and heterogeneity at 1303. The revertant at 1050 was also found in this sample. Three samples (recovered from high dose-inoculated mice) showed sequence heterogeneity at position 2788 leading to a deduced mix of G/S at residue 924 (i.e., a mix of revertant wt/inoculated sequences); in these three samples the position 1303 did not change. Finally, six of the samples (4 low, 2 high) maintained the original inoculated sequence (mutant version: S924 and T1303). No other changes were found in the region analyzed (RdRp amplicon).

These results suggest that replication in immunodeficient animals lacking an efficient innate immune response leads to the rapid reversion and emergence and fixation of new changes likely providing some fitness advantage, and that this evolution was prompter with a wt NSs than with a mutated one.

### Immunogenicity and Efficacy Assays (Vaccination and Challenge) in Immunocompetent Mice

**Figure 5:**
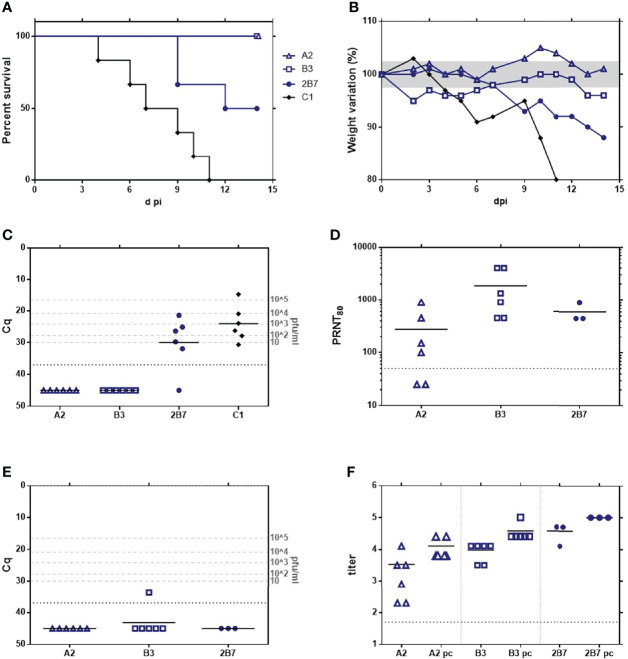
Analysis of the immunogenicity and vaccine efficacy of the rZH548-L mutant viruses in 129 (wt) mice. Groups of 4-month-old mice (n = 6, including male and female equally distributed) were inoculated with 500 pfu of rA2 and rB3 viruses. C1 (rZH548) and 2B7 (rZH548-P82L) were included for a comparison. 3 weeks later survivors were challenged with a lethal dose of 1000 pfu of C1 (rZH548). **(A)** Survival rates after first inoculation. **(B)** Percentage weight variation, geometric mean per group. The area between 97.5 and 102.5% has been shaded. **(C)** Viremia determined by RT-qPCR on EDTA blood samples collected at day 3 pi. Samples giving a Cq (quantification cycle) value under the detection level of the assay (37) are arbitrarily represented as 45. The correlation of Cq data with pfu equivalents (Moreno et al., 2020) is indicated in the right Y axis. **(D)** Neutralizing antibodies at day 12 pi as determined by a plaque reduction assay. **(E)** Viremia determined by RT-qPCR on EDTA blood samples collected at day 3after challenge. **(F)** Antibodies to N protein by ELISA both pre- and post-challenge (small and large symbols respectively).

The immunogenicity of the rescued viruses rA2 and rB3 was then analyzed in a vaccination-challenge assay in 16 week-old, immunocompetent 129Sv wt mice. Groups of six animals of both sexes equally distributed, received IP 500 pfu of viruses obtained after 3 post-transfection passages. The rRVFV-C1 (wt ZH548) was included as a control and, for comparison, we included again the rZH548-P82L virus (2B7). Animals were monitored daily for the development of disease and survival. Blood samples collected at day 3 pi were checked for viremia levels by RT-qPCR, while serum samples collected at 12 dpi were used to analyze seroconversion, both for the development of anti-N protein and neutralizing antibodies (**Figure 5**).

In the group inoculated with the wt control virus (C1 = rZH548), all the animals scored positive at viremia, with Cq values in a range between 14.58-30.61 (mean =25.10). There were no survivors, with the first death occurring on day 4 and the last one on day 11 (MST = 8). As expected, 2B7 showed some attenuation with the first death at day 9, a final survival of 50% of animals and an increased MST to 13 days. Viremia levels in this group were slightly lower than in the control group, with one mouse with a Cq value below the detection level of the assay (Cq=37), and the other 5 in the range of 21.41-31.91. Conversely, the groups inoculated with rA2 and rB3 rendered a 100% survival with all samples scoring negative for viremia (Cq values below the detection level). Anti-virus antibodies, both by neutralization assays (**Figure 5D**) and by anti-N ELISA (**Figure 5F**, small symbols), were detected in all animals, with mean values slightly higher in the group inoculated with rB3, although the difference was not statistically significant (one-way ANOVA). No signs of disease were observed in animals within these groups, except for some weight loss in the first days for 3 out of the 6 animals within group B3 (**Figure 5B**).

After proving the induction of anti-RVFV immunity, animals were exposed to a lethal challenge with 1000 pfu of the virulent rZH548 (C1, wt) and controlled for 18 days. At this time point, taken as the end of the experiment, all the animals had survived. One only sample, from one animal within group B3, scored positive in viremia on day 3 although with a low viral load (Cq = 33.69, **Figure 5E**). Serum samples collected at day 14 post-challenge revealed an increase in antibody levels, both neutralizing and anti-N, thus verifying that the animals had been exposed to viral challenge (**Figure 5F**, large symbols).

These results show that the two amino acid changes introduced in the viral RdRp (G924S and A1303T) drastically decrease the virulence of the wt rZH548-RVFV for immunocompetent mice, with 100% of survivors in the absence of signs of disease and undetectable viremia levels after inoculation with 500 pfu. The weight loss observed after inoculation with rB3, together with the slightly higher antibody titers at day 12 could be indicative of a (non-surprising) higher level of replication of this virus that carries changes only in the L-protein, although differences were not statistically significant. Nevertheless, both rA2 and rB3 viruses afforded equivalent good results in terms of inducing protective immunity, supporting their vaccine potential. Furthermore, in these animals no viral RNA could be detected, indicating low levels of viral replication and, therefore, diminishing the risk of reversion found in infections carried out in IFNAR KO mice.

## Discussion

In this work, we show that the substitution of only two amino acids, G924S and A1303T, in the polymerase of a virulent RVFV (rZH548) is enough to strongly attenuate the virus without loss of protective immunogenicity, therefore supporting their application for safety improvement of a live attenuated vaccine (LAV). Targeting key positions in viral RNA polymerases is a strategy suggested for the rational design and improvement of LAVs that has been proved successful for several virus families (Lee et al., 2016; Rai et al., 2017; Yeh et al., 2020; Mori et al., 2021). These changes may however have adverse effects on the polymerase functionality leading to some fitness cost that can be too disadvantageous for virus replication in the host and for vaccine production. Subsequently, combination with compensating changes or reversion to the original sequences may occur (Domingo et al., 2012; Domingo and Perales, 2019). Therefore, understanding the mechanisms of action and their dynamics is important when considering the introduction of these potential attenuating changes in a live vaccine candidate.

**Figure 6:**
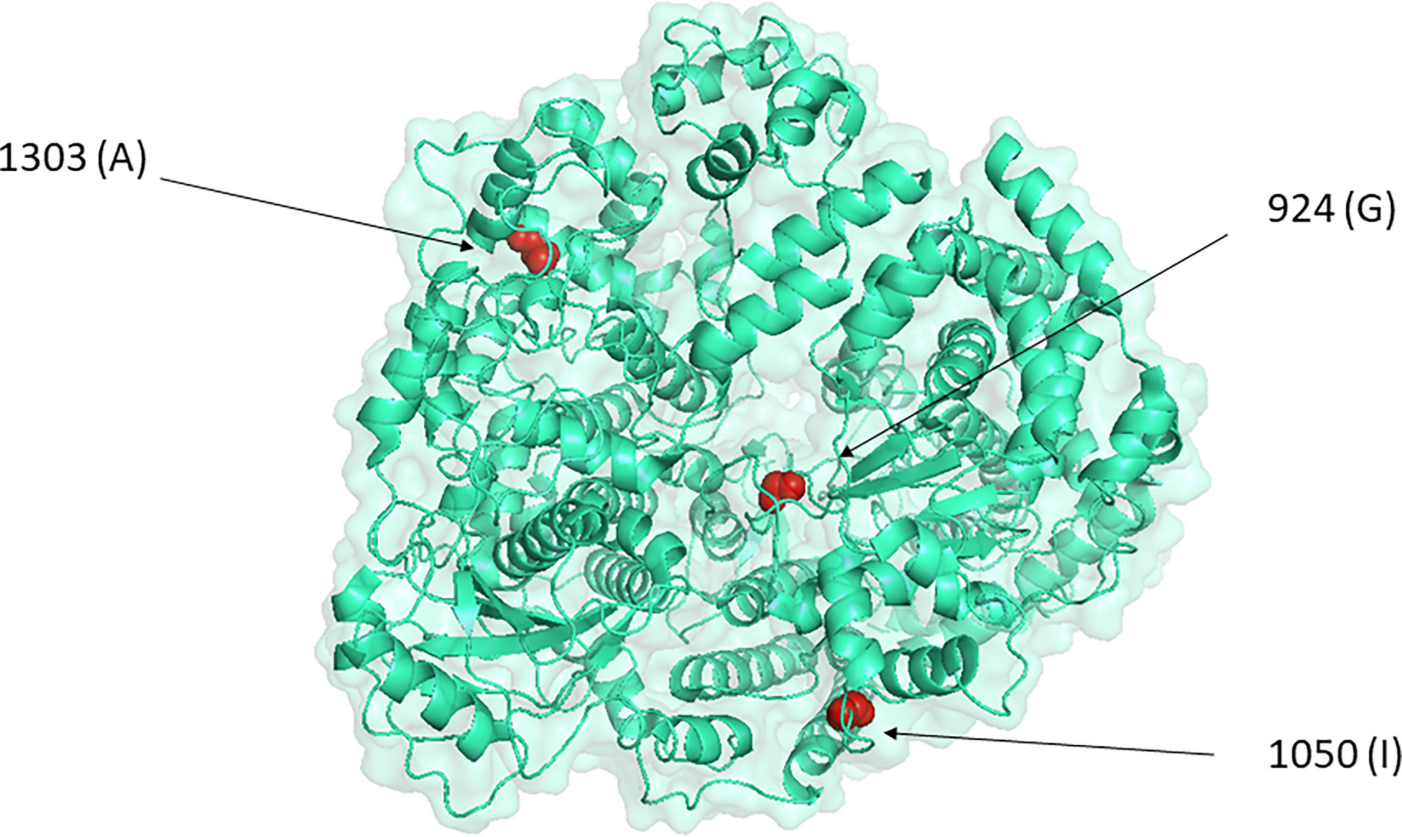
Location of the three positions under study in the representation of RVFV L-protein by PyMOL, as defined by the cryo-EM model (accession code 7EEI, (Wang et al., 2021).

The two changes mentioned appeared in a RVFV variant selected in the presence of the mutagenic drug favipiravir, named as 40Fp8, which was found to be highly attenuated *in vivo* (Borrego et al., 2019; Borrego and Brun, 2020). This variant displayed 24 amino acid substitutions, 7 of them within the L-protein, the viral polymerase. Here we focused on those in residues 924, 1050 and 1303 because of their location in the catalytic core of the RNA-dependent RNA polymerase (RdRp). In particular, residue 924 is located within motif F in the RdRp core, a motif involved in the binding of the incoming rNTP (te Velthuis, 2014; Ferron et al., 2017; Wang et al., 2021) as well as in the interaction of favipiravir with the viral polymerase. A small number of viruses has been isolated with altered sensitivity to favipiravir that share the substitution of a conserved K within the F-motif, associated to a cost to viral fitness that can be restored by accompanying mutations, and these changes affect the polymerase activity and maybe also its fidelity (Delang et al., 2014; Abdelnabi et al., 2017; Goldhill et al., 2018). Since G924 is structurally located between the two key positive-charged residues K918 and R926 conserved in F-motifs of RdRps from different RNA-virus families (te Velthuis, 2014; Peng et al., 2021), it seemed reasonable to attribute a significant role to the G924S substitution and its combination with I1050V and/or A1303T. The recent cryo-EM model of RVFV RdRp locates residue 924 in the fingertips, residue 1050 within the finger node and 1303 within the thumb (Wang et al., 2021), but no precise locations or interactions nor an obvious structural/physical connection among the three residues provide a clue about their role (**Figure 6**).

To get some insight into the effect of these substitutions on viral growth, we rescued rRVFVs-ΔNSs::gfp carrying the 3 selected changes in different combinations and tested them in cell culture and *in vivo*. Not surprisingly, substitutions G924S and A1303T rendered viruses with some growth deficiency, hardly detected by cpe, with short viral yields and tiny turbid plaques. In contrast, the substitution I1050V seemed to enhance growth, with viruses carrying this change (either single, double or triple mutants; **Figure 1**) grow to titers even higher than the wt. When tested in immunodeficient mice, SM#2 was the only rescued virus with a pattern undistinguishable from the wt virus, while all the other combinations conferred some level of attenuation. These results suggest that the change at 924 displayed by 40Fp8 allowed its growth in the presence of favipiravir with a likely fitness cost compensated by the change at 1050, while we cannot conjecture on the role of the change at residue 1303, maybe functionally related, or appearing simultaneously by chance in the mutagenized 40Fp8 and contributing to its features.

For a live RNA virus vaccine a reduced variability would pose a further advantage in terms of diminishing the risk of reversion when replicating *in vivo* and also in manufacturing procedures (Vignuzzi et al., 2008; Lokugamage and Ikegami, 2017; Ikegami, 2021; Mori et al., 2021). The analysis of nucleotide diversity as a measure of genetic variability after a short number of replication cycles of the populations of ΔNSs_L mutants suggested that these changes might be affecting the fidelity of the corresponding polymerases, leading to a small variability reduction for almost all mutant combinations (**Figure 1C**). Even though small, it is to note that the L-mutant viruses were originated from DNA plasmids and the analysis was performed after only 4 passages after transfection. However, results provided by the growth curves in the presence of antiviral drugs of viruses A2 and B3, both carrying a mutant RdRp [G924S and A1303T], would indicate that these changes do not affect the fidelity of the mutant polymerase, since these viruses did not show an altered sensitivity to ribavirin. The notion that high-fidelity viruses are resistant to ribavirin and other nucleoside analogs as proof of a higher selectivity for nucleoside incorporation comes mainly from studies on ribavirin-resistant viruses (Pfeiffer and Kirkegaard, 2003; Arias et al., 2008; Coffey et al., 2011; Cheung et al., 2014; Van Slyke et al., 2015), but CVB3 mutants with altered susceptibility to favipiravir but not to ribavirin have been found to be affected in polymerase fidelity (Abdelnabi et al., 2017). Virus fidelity is a complex subject involving other aspects such as processivity or kinetics and may be affected by environmental conditions (Kautz and Forrester, 2018). In this sense, it should be considered that the growth curves in the presence of antiviral drugs were performed with RdRp-mutant viruses in a wt background while the nucleotide diversity analyses were carried out with (ΔNSs::gfp)- RVFV mutants. The NSs deleted-gfp expression allowed an easier rescue of mutant viruses by means of the fluorescent reporter, but it became a double-edged sword for some studies, impairing a fine quantification as well as interpretation of attenuation results. Although the analyses include the corresponding internal control, we cannot assure that the absence of NSs causes also some bias on the features assayed (see further).

Even though more accurate studies on activity and fidelity of the mutant polymerases remain to be done, the main finding of our work is the pivotal role on virulence exhibited by the combination of changes G924S and A1303T, suggesting that their additional introduction in a live-attenuated-vaccine could improve its safety. The (ΔNSs::gfp)-L mutant carrying them, DM #5, offered a longer survival in the extremely sensitive IFNAR KO mice. In a wt rZH548 background, RdRp [G924S and A1303T]-mutant viruses induced protective immune responses in inoculated mice in the absence of viremia and signs of disease, thus confirming that these changes rendered an attenuated virus. Undetectable viremia suggested a low level of replication, thus decreasing the risk of reversion and the chance of transmission. However, these viruses, even the one carrying a third attenuating determinant (NSs [P82L]; (Borrego et al., 2021) were still virulent for immune deficient animals, revealing that the high attenuation displayed by 40Fp8 is achieved by further changes in other proteins or positions that remain to be identified.

In addition, the rescued virus rA2 carrying the triple combination RdRp [G924S and A1303T] and NSs [P82L] revealed a surprising new advantage in terms of genetic stability. When grown in cell culture for a short number of passages virus rB3 changed to gain the wt sequences, while no changes were observed in virus rA2. IFNAR KO mice experiments corroborated these findings, with a higher number of original sequences in samples collected from rA2 inoculated-animals (**Table 3**). In addition, these studies support that A129 mice may provide a good model to explore the pathways of virus evolution *in vivo*. Since both rA2 and rB3 viruses originated from the same plasmid carrying the L-mutated segment (so possible input wt traces would be the same), these results suggest that an altered NSs expression, in this case provided by the P82L-mutated-protein, can affect the chance or efficiency of replication and reversion, an effect already pointed out in NSs-deleted mutants (Lokugamage and Ikegami, 2017). On the other hand, sequences recovered from this experiment support our hypothesis that changes S924 and T1303 entailed some kind of growth disadvantage driving their revertion or requiring a compensation by an advantageous change like that on residue 1050. Should these two changes be considered as attenuating determinants for a safer live vaccine, a strategy to prevent their reversion could be explored. The use of codons requiring more than one only nucleotide change or a transversion instead of a transition would probably decrease their chance to revert to the original sequence.

In summary, we have shown that the combination of two single substitutions, G924S and A1303T, in the L-protein of the virulent RVFV rZH548 leads to viral attenuation without affecting the capacity to induce an immune protective response. Rapid reversion in cell culture and in the absence of efficient immune responses suggest that these are key positions in the viral cycle. The combination with a further attenuating determinant such as NSs[P82L] provides promising results in terms of increased attenuation and higher genetic stability. Although the mechanisms underlying the effect of these changes on polymerase activity and thus in viral attenuation remained to be elucidated, our data illustrate the risk of reversion during vaccine production or when inoculated *in vivo* for RNA viruses-LAVs based on single mutations, and highlight the need for accurate studies on the mechanisms and dynamics of such changes before considering their inclusion as attenuating determinants.

## Data Availability Statement

The datasets presented in this study can be found in online repositories. The name of the repository and accession numbers can be found below: NCBI; PRJNA807489.

## Ethics Statement

The animal study was reviewed and approved by Animal Care and Biosafety Ethics’ Committees of INIA and Comunidad de Madrid.

## Author Contributions

Conceptualization, BB and AB. Methodology, BB, SM, NL, ÁL-V, JN, and AB. Formal analysis, BB, SM, ÁL-V, JN, and AB. Resources, SM and FW. Supervision, BB and AB. Writing—original draft, BB. Writing—review and editing, BB, SM, ÁL-V, JN, FW, and AB. Funding acquisition, BB and AB. All authors contributed to the article and approved the submitted version.

## Funding

This work was supported by grants S2013/ABI-2906 (PLATESA), P2018/BAA-4370 (PLATESA2) from Comunidad de Madrid/FEDER and AGL2017-83326-R from Ministerio de Economía y Competitividad and PdC2021-121717-I00 funded by MCIN/AEI /10.13039/501100011033 and by Unión Europea Next Generation EU/ PRTR. AL-V has a pre-doctoral fellowship FPI 2017, Ministerio de Ciencia, Innovación y Universidades from the Spanish government.

## Conflict of Interest

INIA has filed an international patent application (code # WO2021/245313A1) for 40Fp8-based RVF vaccines.

The authors declare that the research was conducted in the absence of any commercial or financial relationships that could be construed as a potential conflict of interest.

The handling editor MS declared a past co-authorship with the author BB.

## Publisher’s Note

All claims expressed in this article are solely those of the authors and do not necessarily represent those of their affiliated organizations, or those of the publisher, the editors and the reviewers. Any product that may be evaluated in this article, or claim that may be made by its manufacturer, is not guaranteed or endorsed by the publisher.
